# Flaws in advance directives that request withdrawing assisted feeding in late-stage dementia may cause premature or prolonged dying

**DOI:** 10.1186/s12910-022-00831-7

**Published:** 2022-10-06

**Authors:** Stanley A. Terman, Karl E. Steinberg, Nathaniel Hinerman

**Affiliations:** 1 Caring Advocates, Sausalito, CA USA; 2 California State University Shiley Haynes Institute for Palliative Care, San Marcos, CA USA; 3 Department of Theology and Religious Studies and School of Nursing and Health Professions, University of San Francisco, San Francisco, USA

**Keywords:** Advanced dementia, Late-stage dementia, Advance directives, Advance care planning, End-of-life decision-making, Suffering in dementia, Ceasing assistance with oral nutrition and hydration, Voluntarily stopping eating and drinking, Paternalism

## Abstract

**Background:**

The terminal illness of late-stage (advanced) Alzheimer’s and related dementias is progressively cruel, burdensome, and can last years if caregivers assist oral feeding and hydrating. Options to avoid prolonged dying are limited since advanced dementia patients cannot qualify for Medical Aid in Dying. Physicians and judges can insist on clear and convincing evidence that the patient wants to die—which many advance directives cannot provide. Proxies/agents’ substituted judgment may not be concordant with patients’ requests. While advance directives can be patients’ last resort to attain a peaceful and timely dying consistent with their lifelong values, success depends on their being effective and acceptable. A single flaw can provide opponents justification to refuse the directive’s requests to cease assisted feeding.

**Aim:**

This article considers 24 common advance directive flaws in four categories. *Process flaws* focus on how patients express their end-of-life wishes. *Content flaws* reflect drafters’ selection of conditions and interventions, and how they are described. *Inherent flaws* can make advance directives unacceptable to authorities concerned about premature dying. *Strategies* are needed to compel physicians to write needed orders and to prevent third parties from sabotaging these orders after they are  implemented. The article includes excerpts from “dementia-specific” directives or supplements that exemplify each flaw—mostly from the US and Europe. No directive critiqued here included an effective *strategy* to resolve this long-debated bioethical conflict: the past directive requests “Cease assisted feeding” but the incapacitated patient apparently expresses the desire to “Continue assisted feeding.” Some opponents to the controversial request, cease assisted feeding, use this conflict as a conceptual wedge to practice hard paternalism. This article proposes a protocol to prevent this conflict from emerging. These strategies may prevent authorities from requiring patients to fulfill *authorities’* additional clinical criteria as a prerequisite to honor the requests in patients directives.

**Conclusion:**

This critique of flaws may serve as a guide to drafting and to selecting effective and acceptable advance directives for dementia. It also poses several bioethical and clinical questions to those in authority: Does your paternalistic refusal to honor patients’ wishes respect their self-determination? Protect vulnerable patients from harm? Force patients to endure prolonged suffering? Violate the principles of bioethics? Violate the very foundation of patient-centered care?

## Background: advanced dementia can be a cruel, burdensome, and prolonged terminal illness

 More than half of people fear the diagnosis of dementia and 62% think it means “life is over” (UK) [1]. People complete advance care planning in order to avoid prolonged dying in advanced (late-stage) dementia. They dread this cruel and burdensome terminal disease because—regardless of the specific diagnosis of dementia—in the late or advance stages, it causes loss of memory; change in personality; decreased cognitive functioning; inability to communicate; embarrassing and sometimes dangerous behaviors; and dependence on others for most, and ultimately all, personal care. Less well-recognized is that patients may suffer from undetected (and therefore untreated) severe physical pain after they lose the ability to complain. Dementia also imposes huge emotional, physical, and financial burdens on loved ones, many of whom continue their sacrifice after patients no longer can either recognize or enjoy them.

Traditional advance directives typically omit suffering from sources that cannot be observed contemporaneously so that many clinicians say, “She is just sitting there.” Yet three sources, for example, can cause non-observable severe suffering: disruption of life narrative; leaving undesired, tarnished memories with surviving loved ones; and cognition so impaired that patients can no longer interact meaningfully with other human beings that leads, in turn, to extreme social isolation and existential suffering (since all their human relationships will have died). Unfortunately, none of these sources of suffering can be treated. Advanced dementia patients may also experience emotional suffering that results in withdrawal that does not present a management problem, and therefore may not be treated.

The problem is huge: One-third of people over 65 now die *with* dementia [2]. By Mid-Century, as many as 1 of 12 people over 65 may be living in an advanced stage of dementia [3].^1^

This article focuses on advance instructional health care directives (henceforth, *directives*), which are often referred to as living wills or advance decisions (UK). The article also uses *directives* when critiquing instructional supplements. (A companion article presents the problems with asking surrogates for their substituted judgment to fulfill patients’ end-of-life goals.^2^) This article first explains why directives can be the last resort to avoid a prolonged dying in advanced dementia. Then it focuses on 24 common flaws—one flaw at a time—that may prevent directives from succeeding. “Success” is defined as leading future treating physicians to write orders that conform to the directive’s specific requests. Most examples are drawn from US drafters because many other countries encourage physician–patient shared decision-making conversations rather than completing template forms. Yet flaws have no borders and what is learned from flawed templates can be applied to clinicians’ conversations, so they can better ask the right questions. The article ends on an optimistic note, with two successful directives. The Appendix presents the flaws in one popular directive.

### Ways to reduce suffering in advanced dementia are limited

New medications to prevent dementia, postpone onset, or slow its progression have not been definitively proved to be efficacious [4, 5].^3^

Medical Aid in Dying, which term could be considered a euphemism for physician-assisted suicide, is not generally available to patients living with dementia because they cannot meet two required criteria: capacity and likely to die within six months (in about 10 US jurisdictions).

While selecting a proxy/agent as a surrogate decision-maker is important [6–9], physicians, administrators, and judges may not follow their instructions unless they present clear and convincing evidence that patients want to forgo all life-sustaining interventions. Example: a court denied the request of the husband/proxy/agent for Norah Harris [10], which was consistent with the 1990 U S Supreme Court ruling in *Cruzan* [11] that affirmed states’ right to insist that “No one may refuse treatment for another person, absent an adequate living will or clear and convincing, inherently reliable evidence” [12]. The challenge of poor concordance between proxies/agents’ and patients’ requests is well-known [13].^4^ Finally, while most proxies/agents care deeply about their relatives’ well-being, few have sufficient experience, training, knowledge, and arguing skill to overcome the resistance of physicians and administrators to persuade them to honor patients’ controversial requests. One sad example is Margaret Bentley, who was force fed for five years [14, 15].

On the other hand, following proxies/agents’ healthcare instructions may not reflect patients’ requests. A review by Dening et al. [16] concluded that “a key barrier” to successful advance care planning for persons with dementia “is the potential for proxy decision-makers to influence end-of-life care that may not reflect the wishes of the person with dementia.” Dening also used a nominal group technique with UK subjects that showed, “Many carers believe that they are making decisions in accordance with the wishes of their family member [but] may be making choices…bound up with their own experiences [that are] not concordant with those of the person with dementia had they retained capacity” [17].

If advanced dementia cannot be prevented, postponed, or adequately treated; if advanced dementia patients cannot qualify for Medical Aid in Dying; and if consistent testimony of loved ones, proxies/agents, and others can be judged *not* clear and convincing or is wrong by not being concordant with patients’ requests—then attaining a peaceful and timely dying will depend on patients’ directives. But directives must not only be accurate; they must also be clinically effective and acceptable to those in authority*.* By themselves, directives may not be successful. Patients may need additional strategies to persuade physicians to write and implement needed orders and to prevent third parties from subsequently sabotaging these orders.

In their 2004 article, “Enough: the failure of the living will” [18], Fagerlin and Schneider asked: “How can anything [living wills] so intuitively right be proved so infuriatingly wrong?…The failure to devise workable forms is not a failure of effort or intelligence. It is a consequence of attempting the impossible.” Despite such pessimism, at least a dozen new or revised “dementia-specific” directives were drafted in the US between 2014 and 2020. This article critiques most of them.

### Challenges of directives for late-stage dementia are formidable

Many advanced dementia patients can survive for years without high-tech life-sustaining medical treatment. In lay terms, they have “no plug to pull.” To attain a timely dying based on patients’ lifelong critical wishes, ceasing assistance with oral nutrition and hydration (henceforth, *assisted feeding*, with the understanding that this term always includes assisted oral hydrating) is both peaceful and feasible since most advanced dementia patients eventually become dependent on carers (caregivers) for assisted feeding [19, 20].

Directives that request ceasing assisted feeding are controversial. To some, they are counter-cultural or immoral. Our food-obsessed culture equates food with love, and feeding with loving.

Many feel it is cruel to cease assisted feeding from patients who *appear* willing to open their mouths and swallow what others put in. Yet these observers often fail to consider that such behavior in a terminal illness can result from a reflex, and that continuing assisted feeding can merely prolong the process of dying and its associated (perhaps undetected) suffering.^5^

The most relevant bioethical perspective of this article is the patient-centered aspiration stated in Opinion 2.20 of the American Medical Association’s Code of Ethics:

The social commitment of the physician is to sustain life and to relieve suffering. Where the performance of one duty conflicts with the other, the *preferences of the patient* should prevail [21].

### Aims of this article

This article identifies 24 types of flaws in directives—*not* to critique individual directives, but to increase readers’ awareness of common flaws that forthcoming directives and clinicians’ shared decision-making conversations should avoid. The article supports its critical review by evaluating directives *on their face*, by authors’ clinical experiences, and by literature reports—since it will take years until sufficient clinical outcome data are available to analyze if, by then, currently available directives will have been successful. Some bioethical issues are beyond the scope of this article,^6^ such as directives for people who want to live as long as possible [22].^7^

In order to reduce the intensity of controversy surrounding a physician order to cease assisted feeding, this article insists directives fulfill two clinical/ethical standards: (A) persons engaged in advance care planning (henceforth, *planning principals*) must judge which specific conditions would cause “severe enough suffering” to want to be allowed to die based on their personal values; and (B) the ultimate cause of death for the incapacitated patient, whom the planning principal may someday become, must be the underlying disease, although the timing may be influenced by when treating physicians and proxies/agents decide to *withdraw* assisted feeding.

### The dire consequences of not honoring directives

Two types of dire consequences from not honoring directives are: (A) prolonged dying associated with severe suffering, and (B) premature dying if patients act on their “Dementia Fear.” To explain the latter: if patients cannot trust others will honor their wishes, they may worry about waiting too long to orchestrate their dying before they lose capacity after which they will not be able to avoid years of misery in advanced dementia. Example: *Still Alice* [23] was not able to follow her previously written directions by the time she reached her criteria to want to die. Attorney bioethicist Dena Davis [24] recommended committing preemptive suicide before losing capacity as the only certain way to avoid advanced dementia. But suicide can sacrifice years of reasonably good living. This article encourages completing directives that avoid common flaws—so patients can feel confident that others will honor their directives, so they will not even consider hastening their dying until their suffering has become severe.

### Successful directives must provide adequate answers to two questions

The instructional part of all instructional directives follows the format, *If…Then*:

**If** I reach [a certain condition], **then** I want [a specific intervention].

Answers to the *When Question* (the *If*) must *adequately* indicate when to cease life-sustaining treatments without ambiguity or conflict. Answers to the *What Question* (the *Then*) must be *effective* and *acceptable.* Medical dehydration is effective since patients usually die within two weeks. The dying process has also been shown to be peaceful; for example, a study where hospice nurses observed alert patients [25]. To be *acceptable*, authorities in power must be able to view patients’ requests to cease assisted feeding as clinically appropriate, legal, ethical, moral (culturally correct), and if possible, consistent with the teachings of the authority's religion.

### One general flaw is to evaluate directives based on only their descriptive definition

A descriptive definition of a directive:

An instructional directive is a form that a planning principal completes in the event of future incapacity to inform physicians and other health care providers^8^ about their treatment preferences if the planning principal were to reach future specified clinical conditions.

An operational definition of a directive {where the non-Italicized word indicates the important additions}:

An instructional directive is a form that a planning principal completes in the event of future incapacity *to provide evidence* that their proxies/agents can use to persuade their treating physicians and other health care providers *to promptly honor* their treatment preferences, if the planning principal were to reach future specified clinical conditions, *by writing or carrying out conforming medical orders*, which third parties let stand.

A descriptive definition of a directive may be judged successful upon its completion, but still fail to fulfill the operational definition in the future, when it is implemented. The promise of advance directives to fulfill patients’ self-determination requires concordance between planning principals’ requests during advance care planning and the implemented medical orders in the future.

An example: A Nevada law [26] strives to answer the *What Question,* but *not* the *When Question*, a flaw that makes it unlikely to succeed. While the statute legalizes ceasing assisted feeding, the vague *When* criterion, “You can…state what you want to happen if you get very sick and are not likely to get well,” prompted Thaddeus Pope (among others) to ask: “When do we stop offering food and fluids? How do we ascertain the satisfaction of any ‘trigger’ conditions the patient specified?” [27]. Here, Pope implicitly doubted the ability of planning principals to write their own statements that will be persuasively clear and convincing. Writing one’s own flawed directive partially contributed led to an infamous case in the Netherlands (discussed below) in the flaw category, “Descriptions are Ambiguous, Vague, or Inconsistent.”

### Some drafters of directives admitted their success is uncertain

Jonathan Patterson, an attorney at Compassion & Choices wrote, “Even though you put your wishes there, it doesn’t mean a medical professional will honor it” [28]. Judith Schwartz, a clinician at End of Life Choices New York, stated about the “Advance Directive for Receiving Oral Foods and Fluids in the Event of Dementia” [29] (henceforth, *NY Directive*): “Please note that this Directive has not received judicial review.” Drafters of Compassion & Choices’ “Dementia Values and Priorities Tool” [30] (henceforth, *C&C Tool*) advised, “Doctors and health care advocates worry that they will be charged with elder abuse.” Drafters of the “Supplemental Advance Directive for Dementia Care” [31] (henceforth, *SADD*) admitted, “There is very little legal guidance in state law on whether a healthcare provider [is] legally required to honor your VSED request.”

*Conclusion* The brief review above leads to this recommendation: planning principals must devote sufficient effort to complete directives whose instructions are clear and convincing, and that adds strategies to make the directive not only durable but irrevocable, so it can stand on its own rather than depend on their physicians or proxies/agents to direct the end-of-life care that patients desire. This challenge, most commonly but not exclusively, affects patients living with advanced dementia.^9^ The rest of this article analyzes flaws in directives that must be avoided so that advance care planning can lead to effective, acceptable advance directives.

## Four types of flaws

This article defines “flaw” as an element in an instructional directive or supplement that renders the directive ineffective or unacceptable, or unable to overcome common clinical or legal challenges, and thereby thwarts planning principals from attaining their end-of-life goals.

The article has two parts. Part ONE considers flaws as capacitated planning principals complete their directives, which are:

Type I flaws (1 to 6) focus on the *process*, the actions of planning principals, as they complete their directives.

Type II flaws (7 to 13) focus on the *content*, such as which clinical conditions and interventions the drafters of directives included, and how they described them.

Part TWO considers flaws as physicians and others either do or do not implement previously completed directives after the advanced dementia patient has lost capacity:

Type III flaws (14 to 19) explore two types of *inherent* flaws: directives that have ineffective interventions, and directives that opponents consider unacceptable.

Type IV flaws (20 to 24) considers the flaw of *omitting strategies* designed to compel physicians to write orders to honor patients’ requests, or designed to prevent third parties from sabotaging physicians’ written orders.

## PART ONE: Flaws committed as planning principals complete their directives

### Example: Margaret Bentley’s flawed directive failed to accomplish her end-of-life goal

Early in her career as a nurse, Margaret Bentley cared for patients as they (very) slowly died from advanced dementia. This experience made her certain she wanted to avoid prolonged dying if she ever received the diagnosis of dementia. While well-informed about the disease, she did not have adequate information about advance care planning. The main reason was that in 1991, British Columbia had no directive statute, so she had to compose her own. She also designated her husband and her daughter, a nurse, as surrogate health care decision-makers. Both promised their best efforts to not let her endure a prolonged dying in advanced dementia.

Years later, despite their persistent efforts, husband and daughter failed to convince physicians, administrators, and judges to honor Margaret’s directive. The consequence was five years of force feeding and suffering that included pain marked by moaning during transfers between bed and Geri chair due to severe joint contractures. Sadly, Margaret suffered from slow but relentless starvation. Before she died, she resembled a holocaust victim [15].

How did this happen? In striving to fulfill their perceived role “to protect” their patient, nursing home administrators refused to let stand, the physician’s initial order to cease assisted feeding. Later, administrators requested an elder abuse investigation and obtained a restraining order to prevent Margaret’s husband and daughter from taking her home, where she might have been allowed to have a peaceful and timely dying. In court, an unqualified “expert” gave unopposed testimony based on a few observations of Margaret’s *apparent* cooperation with assisted feeding and interpretated her behavior as preferring sweet foods. This observation was used to support the contention that Margaret had sufficient capacity to “change her mind” and now wanted assisted feeding to continue.

How could administrators and judges override the authority of Margaret’s directive and proxies/agents? Her directive had at least four flaws: (A) It did not specify “oral” to refuse food and fluid by mouth. This flaw permitted a judge to base his ruling on the presumption that she wanted to refuse only tube-feeding. (B) She requested “to be euthanized” if she could not recognize family members, which was illegal in Canada (at the time) and likely alarmed administrators. She should have instead made this request: “If and when euthanasia becomes legal.” (C) It omitted strategies designed to overcome third parties’ allegations that she changed her mind to want assisted feeding to continue, as well as strategies designed to prevent a potentially prolonged adult protective service investigation. (D) It omitted strategies designed to make her request to refuse assisted feeding irrevocable, regardless of how others interpreted her current behavior.

Three important conclusions emerge: (A) A single flaw can prevent a directive from being operationally successful. (B) By itself, a flawless instructional directive may not succeed without additional strategies. (C) Flaws can lead to either prompt or delayed refusals; both can deny patients a timely dying. Prolonged conflicts can deny patients’ wishes, even if resolved in the patients’ favor. The clinical statement, “To delay is to deny,” is similar to the legal statement, “Justice delayed is justice denied.”

Directives can be considered patient decision aids since their function is to inform patients of their possible future conditions and treatment options. Directives facilitate making advance treatment decisions; they are conditional consents for accepting or refusing future treatment. If flawed, those in power may refuse to honor a directive, which they can justify by alleging it would cause premature dying that is not in their patients “best interest.” The result can be years of prolonged dying with associated suffering.

## Type I flaws: the *process* by which planning principals complete advance directives

1. Doesn’t Allow Discriminating Refusal of Oral Nutrition = DADRON (0) [32],^10^

The initial act in the process of advance care planning is to select a directive. Traditional advance directives rarely offer the intervention, “Cease oral assisted feeding and hydrating.” For example, in the UK, Compassion in Dying offers an Advance Decision Pack that suggests this wording for refusing future treatment: “I understand life-sustaining treatment includes but is not limited to CPR, clinically assisted nutrition and hydration, artificial or mechanical ventilation and antibiotics for life-threatening infections.” It also offers this guidance: “You cannot use an Advance Decision to refuse basic care that keeps you clean and comfortable” [33]. The challenge: some authorities argue that assisted feeding provides comfort.

The *Five Wishes* form [34], is even worse. It is subtly coercive in continuing assisted feeding by including a statement for which it offers planning principals’ no choice: “I want to be offered food and fluids *by mouth*, and kept clean and warm.”^11^ The form provides no way to reject either the entire request; or better, to reject only “offered food and fluids by mouth” while retaining the request (to be) “kept clean and warm” (which everyone wants). Unless modified, this directive cannot help patients fulfill the goal of avoiding a prolonged dying in advanced dementia. While introduced in the United States, there are 40,000 partner organizations that distribute *Five Wishes* worldwide. Over 40 million copies are in circulation. Patients and family members who feel they can depend on *Five Wishes* to direct the care they want in the last chapter of their lives, and who expect to thereby be able to die how and when they want, may be living with a false sense of security.

All the above forms could lead to an unwanted, prolonged dying.

The C&C Tool [30] facilitates choosing the option, cease assisted feeding, if the patient reaches any one of seven conditions. The tool provides no way for planning principals to reject one or more of its additional seven conditions; for example, “I have to be talked into eating or drinking,” and “I do not accept food without prompting.” Some patients may want continued feeding for some of these seven conditions so they could still live. If so, the tool (directive) could lead to an unwanted, premature dying.

2. Descriptions of Interventions and Conditions Not Understandable = DICNU (0)

The NY Directive [29] states it “will be of use for those in the earliest stages of dementia.” Yet its readability [35] is Grade 14 reading comprehension [36], which is too difficult to “be of use.”^12^ Opponents of ceasing assisted feeding could argue that few early-stage dementia patients have sufficient cognitive ability to comprehend text at Grade 14; hence, the NY Directive is clinically *unacceptable*. In addition to providing opponents a justification to *not* honor the directive, this flaw can provide the basis to persuade a judge that the early-stage dementia patient likely did not understand what they signed and therefore would likely experience an unwanted, premature dying.

3. Provides Inadequate Informed Consent = PIIC (8)

The NY Directive’s [29] answer to the *When Question* is: “By ‘advanced Alzheimer’s disease,’ I mean stage 6 *or* 7 (moderate to severe) of the Functional Assessment Staging Tool (FAST).” But the NY Directive neither cites nor lists the conditions from any specific version of FAST. (This article considers the conditions from two versions, below.) Opponents can argue that the NY Directive does not adequately inform planning principals as it asks for their conditional consent to cease future assisted feeding, which is ethically *unacceptable* and arguably, could lead to premature dying.

4. Presents Other Conditions Inconsistently = POCI (7)

To answer the *When Question*, the online C&C Tool [30] generates a printable display that reflects planning principals’ choices for 15 conditions. But the C&C Tool has a second set of seven (“informational”) conditions that pop up in a temporary online window, but *only* if planning principals click an optional link.^13^ Unfortunately, the printed form does *not* include these seven conditions.

Opponents can argue in two ways. For planning principals who *did* read these seven conditions, the printed directive does *not* completely reflect their advance treatment decisions. For planning principals who did *not* read these seven conditions, the directive did *not* inform them adequately. Both are ethically unacceptable. Both give opponents justification to refuse to honor the directive based on following its requests could lead to unwanted, premature dying.

5. Doesn’t Offer Workable Irrevocability = DOWI (0)

To be successful, directives must be durable at least; irrevocable, at best. Several European countries consider directives legally binding; but in practice, directives may not be clinical binding. A few examples from a 73-page Belgian report [37]: In Austria, “A binding advance directive will lose its validity if the patient…shows by his behavior that it is no longer valid.” The problem is that “shows by his behavior” reflects the observing physician’s interpretation, which may not reflect the patients’ intent or planning principals’ requests. Some European countries have no specific requirement of capacity for patients to revoke directives; others are explicitly permissive. In Estonia, “an advance directive can be withdrawn by a person…without capacity”; in Finland, “without full legal capacity” because “a higher level of capacity is needed to write an advance directive than to cancel it”; and in Hungary, “regardless of the patient’s disposing capacity.”

In the Netherlands, “The healthcare provider may depart [from the directive] if [the provider] considers that there are well-founded reasons for doing so” [37]. Physicians are empowered to judge what constitutes “well-founded,” which may not reflect planning principals’ requests. Giving physicians too much power may be dangerous, but Moratti and Vezzoni illustrated how physicians’ judgment may help avoid harm from this flaw. Directives that “set a specific boundary” to forgo life-sustaining treatments can also consider relevant contrary behavior. Example: “patients cannot recognize their own children” but “patients still appear serene and even smile” [38]. Considering only the boundary without relevant exceptions may lead to premature dying.

In the UK, physicians who fail to comply with directives could be subject to civil liability for battery; but directives will not apply if there are reasonable grounds for believing unanticipated circumstances exist or if the patient has done anything else that is clearly inconsistent with the directive. Again, physicians have the power to judge “reasonable grounds” as they interpret their patients’ behavior.

Some directives include leeway statements that indicate how strictly planning principals want their directives followed. At one extreme is the Dartmouth Dementia Directive, Version 32 [39], which has 100% leeway: “This directive is given to provide guidance and not to limit the authority of my [proxy/agent] or my medical providers.” Clinically, providing “guidance” is *not* likely to be strong enough to persuade physicians to write the controversial order, “Cease assisted feeding”; hence, this directive could lead to unwanted, prolonged dying.

Version 33 of the Dartmouth Dementia Directive attempted to change to the other extreme, zero leeway: “I direct my [proxy/agent] and all treating clinicians to follow the instructions I have given above, no matter the current circumstances, and even if I express a different preference at the time a treatment decision needs to be made.” Santulli, co-creator of this directive, referred to this statement as a “Ulysses type clause”^14^ rather than the more commonly used, “Ulysses *contract.”* Perhaps he realized directives are merely unilateral requests—*not* contracts. But Dartmouth’s and similar directives, such as the SADD^15^ merely make meta-requests (requests about other requests) or define the characteristic *durable* that opponents can refuse to honor.

Directives need additional strategies to avoid being revoked—not only by physicians but also by a planning principal’s “future demented self”; otherwise, directives may be ineffective and lead to unwanted, prolonged dying. Strategies designed to overcome this common flaw are considered in more depth in Flaw #23, Undermining Proxies/Agents’ Power, below.

6. Fails to Ask for Verbal Explanations = FAVE (2)

Dutch clinical researchers speculated on why physicians have a low opinion of directives. One possible reason: they are *not* routinely involved in the advance care planning process [40, 41].

Two steps are needed: physician involvement and adequate documentation.

The Institute of Medicine’s (National Academy of Medicine’s) report, “Dying in America” [42], eschewed advance directives that use checkboxes because merely checking a box *cannot* fully reflect the reasoning that planning principals used to make advance treatment decisions. At best, verbal explanations are memorialized on video in response to relevant questions by an interviewing clinician, so they can provide evidence of a planning principal’s capacity, diligence, and deliberation, and remove doubt that another person completed an online computer program or paper form for them. In addition, patients can provide their personal reasons to help proxies/agents persuade future physicians to comply. Interviews can also help demonstrate that patients signed voluntarily, “under no duress, fraud, or undue influence” [43].

## Type II flaws are about *content*: the particular clinical conditions and interventions that the directive’s drafters chose to include, and how they described them

7. Descriptions are Ambiguous, Vague, or Inconsistent = DAVI (3)

Margaret Bentley’s directive was ambiguous [14]. She requested, “No nourishment or liquids.” But to avoid prolonged dying, she needed to be specific, such as requesting, “Stop putting nourishment or liquids in my mouth.” A judge interpreted her request as refusing feeding by tube. This ruling prolonged Bentley’s dying.

Barak Gaster’s dementia-directive’s intervention is vague: “I would not want any care that would keep me alive longer” [44]. {Original emphasis.} “Any care” is *not* likely to persuade treating physicians to write the controversial order, “Cease assisted feeding,” so Gaster’s directive will likely be ineffective and allow prolonged dying.

In the Netherlands, euthanasia is not prosecuted if the act conforms to the “due care” protocol required by the “Termination of Life on Request and Assisted Suicide Act,” which became law in 2002. Much can be learned from the only case (to date) where the treating doctor was criminally prosecuted [45]. Note: although the patient’s directive requested euthanasia rather than ceasing assisted feeding, it is still heuristic. The advanced dementia patient had completed and then revised her Advance Euthanasia Directive without adequate professional input. Unfortunately, it had three potentially inconsistent conditions: (1) “When I am admitted to a nursing home,” (2) “When the quality of my life has become poor,” and (3) “When I consider the time is right for euthanasia.”

After she was admitted to a nursing home, the patient seemed cheerful and happy despite her previous fear about being forced to endure the kind of miserable existence that had long plagued her demented mother. The geriatrician duly heeded independent medical and expert medical consultations and conferred with the patient’s husband and sister in interpretating her directive. But the geriatrician assumed the patient lacked capacity instead of assessing her current capacity and thus did not ask, “Do you want euthanasia now?” (Previously, the patient had repeatedly responded, “Not yet,” to similar queries.) Furthermore, the geriatrician ordered a sedative to be surreptitiously placed in the patient’s coffee to reduce her resistance to euthanasia. The case is now referred to as *Dormicron*— which is the European brand name of the sedative midazolam (Versed).

Investigation and litigation lasted four years and provoked intense legal and public debate. Eventually there were six rulings including two by Holland’s Supreme Court. To prevent recurrence, the Regional Euthanasia Committees (RTE) revised their guidelines by increasing physicians’ diligence and thoroughness before performing euthanasia. The guidelines already required physicians to assess patients’ decision-making capacity just before preforming euthanasia. But the RTE neither required physicians to offer their input as patients completed their directives nor required physicians to review their patients’ directives for ambiguity, vagueness, and inconsistency. Had such assistance or overview been in effect, this long, litigious ordeal might have been avoided. While the geriatrician was not punished or sanctioned, many wonder if her patient’s dying was premature.

8. Opponents Criticize Individual Conditions = OCIC (7)

The flaws below illustrate how critical opponents can judge specific *content* as unacceptable, claim the directive could lead to premature dying, and ultimately result in prolonged dying.

Menzel and Chandler-Cramer’s advance directive [46] (henceforth, *Menzel*) answers the *When Question* (in part) by: “When I…no longer demonstrate enthusiasm or joy in life activities.” This condition may set too high a bar for cognitively impaired patients who typically manifest apathy, anhedonia, and/or depressive symptoms—but still want to live. Opponents can argue that insisting on enthusiasm or joy are unacceptable criteria since they can lead to premature dying.

Opponents could argue that these three criteria in the C&C Tool [30] (and in the similar directive by End of Life Washington [47]) are unacceptable because they may lead to premature dying: (A) “I can no longer communicate with my loved ones through words.” Yet some advanced dementia patients communicate well nonverbally. (B) “I no longer show interest in foods or liquids, and I have to be talked into eating or drinking.” Yet the role of caregivers is to encourage patients to eat and drink. (C) “No interest in foods or liquids” based on observing nonverbal behavior. Yet patients living with Parkinson’s or Lewy Body Dementia often have “masked facies” that prevent them from showing their emotion and interest.

C&C’s Tool [30] stated, “If…I *begin* to experience delirium, agitation or hallucinations, then I want my medical team to provide palliative sedation in order to avoid suffering *until death occurs*.” {Emphasis added.} Opponents could point to “begin” and “until death occurs” and opine that C&C’s Tool is unacceptable since it is biased to hasten patients’ dying. Also, some causes of agitation are treatable so palliative sedation should not be started without a prior trial of symptomatic treatment; otherwise, premature dying is possible.

Opponents can argue that palliative sedation is “slow euthanasia” [48]. This objection may be overcome by requesting “respite sedation,” whose protocol requires physicians to reduce the dose of sedating medications after a few days [49]. After patients regain consciousness, they can decide if their *suffering* has diminished enough to continue conscious living. Since the protocol is clearly focused on reducing suffering rather than on hastening dying, those in power it may view it as acceptable.

9. Doesn’t Insist on Severe Enough Suffering = DISES (3)

As noted above,  the NY Directive [29] is flawed because it inadequately informs planning principals about specific conditions (the “If” conditions that determine *when* to implement a directive). The NY Directive does not indicate a specific FAST version. If the Medical Care Corporation short version is used for Stage 6 [50], the specific conditions will be: patients need help putting on clothes, bathing, and toileting; patients have urinary incontinence and fecal incontinence. Opponents could argue that even if *all* five clinical criteria were fulfilled, suffering would *not* be severe enough for many planning principals to want to die. If this argument were persuasive,^16^ this directive would be unacceptable since it could lead to premature dying.

In contrast, Menzel’s [46] directive does not share this flaw. First, it lists losses of memory and awareness, personality changes, and “behavior symptoms that include suspiciousness, delusions, hallucinations, or compulsive, repetitive behaviors.” Then it requires reaching a *majority* of the clinical criteria in Stage 6 *and* Stage 7 of Reisberg’s original, full FAST [51].

10. Condition Reached; Is Still Content = CRISC (3)

“Loss of ability to recognize loved ones and close friends” is a commonly dreaded condition of advanced dementia. For one directive reviewed here, it is the sole criterion: SADD [31].

Opponents can point out that as the disease progresses, for a while patients may still enjoy “these nice people”—even though they cannot recall their names or explain how they are genetically related.

In general, there is a significant clinical difference between *reaching* a stage or condition *versus* judging a specific condition would cause severe enough *suffering.*^17^ Some point out that using stages or conditions is necessary since advanced dementia patients cannot inform others when their suffering has become severe enough to want to die. But the obvious counterargument is that they also cannot indicate if they still want to live. Thus, dying could be premature. Authorities may thus consider directives that request allowing death as soon as a patient reaches a stage or condition as unacceptable. They could even argue it is morally wrong to arbitrarily impose the death sentence on vulnerable patients just because they have lost capacity to communicate that they still want to live.

11. Condition Reached; Is Possibly Treatable = CRIPT (5)

Agitated and violent patients are potentially dangerous to self and others. Also, their cost of care can be higher. Somatic pain, intense confusion, and deep fear can drive these behaviors if patients have no other way to express their suffering. Opponents could argue that even if the source of pain cannot be determined, it is clinically appropriate to offer them an empirical trial of analgesics. Retrospectively, if analgesics reduce patients’ disruptive behavior without excessive sedation, then it is likely that they had been suffering from pain. Directives whose instructions skip treatment trials are unacceptable because they can cause premature dying.

12. Fallacy of Composition = FALCOM (0)

The “fallacy of composition” occurs by inferring that if something is true of a part, then it must also be true of the whole. This flaw has been an unintended feature of the Principle of Proportionality since Francisco de Vitoria proposed it in the Sixteenth Century [52, 53]. To reach a decision regarding whether there is a moral obligation to continue treatment that could sustain life, de Victoria argued: if the *act of feeding itself* “caused the patient great trouble or almost certain torment,” then refusing it would *not* be considered a mortal sin. Catholics can accept withdrawing and withholding treatments that have become inappropriate due to patients’ condition; but they can never accept the intent to cause a patient to die, even if the motivation is beneficent; that is, to reduce irreversible severe suffering.

Daniel Sulmasy’s comments are on point: “Persons who suffer, suffer as persons in their totality [and] the burdens of the disease itself, not just the burdens of the treatment, [which] count in the proportionality considerations [since] it is generally the suffering caused by the disease, not its treatment, that constitutes the true burden” [54].

This next example is important because of the influence of the promoting organization. AMDA—The Society for Post-Acute and Long-Term Care Medicine [PALTC] has over 5000 physicians and advanced practitioners who care for dementia (and other) patients who reside in skilled nursing and assisted living facilities [55]. AMDA’s members are highly regarded as thought leaders in dementia care.

In 2019, AMDA committed the fallacy of composition by adopting Resolution A19, a new policy recommended by its ethicists: all residents must receive comfort feeding until their behavior indicates signs of *distress* due to feeding regardless of their directive’s requests [56, 57]. A19 thus followed the teachings of Francisco de Vitoria instead of Daniel Sulmasy. The likely consequence is to prolong dying.

13. Omits Conditions Often Dreaded = OCOD (4)﻿

Directives may be flawed by not being comprehensive; that is, by omitting conditions some people dread enough to want to avoid prolonged dying. Advanced dementia patients who suffer from any omitted condition may experience prolonged dying since their directive will *not* be triggered by omitted conditions.

### Two poignant examples: one religious, one humanistic

Sulmasy asked this rhetorical question about a condition where suffering is difficult to observe:

If a person, integrally considered, is in a state in which he or she is deprived of conscious interaction with the physical world, but not yet dead and united with the One, True, and Eternal Source of all life and all goodness—is this person not in a state of suffering? [54]

Sulmasy’s rhetorical question focused on the relationship between persistent vegetative state patients and God. His question can be transposed to relationships between advanced dementia patients and other human beings. Consider an advanced dementia patient who is apparently “just sitting there,” so her suffering is not obvious for whom this question is analogous:

If a person, integrally considered, is in a state in which he or she is deprived of the cognitive capacity to interact with other people and therefore all joy from human relationships—is this person not in a state of suffering?

The second rhetorical question recognizes patients’ severe existential suffering due to all her relationships having died due to the patient’s severe cognitive impairment. Also, suffering is not limited to the patient since her relatives also suffer.^18^ Omitting conditions that cause suffering can make directives ineffective and prolong dying.

## Part TWO: common flaws when implementing completed directives for dementia patients

This part of the article is based on the premise that it is *necessary but not sufficient* to complete a clear and convincing, specific, and comprehensive directive free of flaws. What else is needed? Interventions must be acceptable and additional strategies must be available to compel physicians to write needed orders and to prevent third parties from sabotaging these orders.

### An example: administrators informed a patient they would not honor her completed directive

In 2013, Susan Saran was a top regulator at the Chicago Board Options Exchange. Then she received the devastating diagnosis that changed her life: frontotemporal dementia. “I was put out on disability. I was told to establish myself in a community before I was unable to care for myself” [58]. At 57, she retired and moved to bucolic Ithaca, New York. She purchased a $500,000 residence at the Kendal Senior Living Community where she intended to live until she died. In 2018, she completed the NY Directive [29]. But after she presented it to Kendal’s administrators and they consulted their legal team, they refused to comply. They may have incorrectly believed institutions are required to assist oral feeding—even if patients legally memorialized their specific request to refuse such assistance [59].

Saran wrote, “I didn’t realize I was signing away my right to self-determination,” and, “I was appalled that my future demented self takes precedence over my competent current self.” She was no longer living with a false sense of security such as, “At least you’ll have a plan” [60]. Reality had struck. At least authorities notified her before she lost capacity, so she still had an opportunity to add relevant strategies or to move to another residence. (She chose the latter.)

## Type III flaws are inherent qualities of completed directives that make them ineffective or unacceptable

14. Intervention Not Clinically Effective = INCE (3)

Gaster’s “dementia-directive” [44] offers this intervention, “I would not want any care that would keep me alive longer.” {Original emphasis.} Patients may be forced to wait years until they contract a life-threatening illness for which all concerned could *then* agree not to treat.^19^ Meanwhile, patients may suffer due to refusing treatment that may both reduce symptoms and preserve life. Gaster’s directive may be ineffective since it may not prevent prolonged dying.

15. Intervention Not Acceptable To Authorities = INATA (0)

One of C&C﻿ Tool’s [30] four treatment options are: “Keep me comfortable while stopping all treatments and *withholding* food and fluid so that I can die peacefully.”^20^Opponents can view directives that request *withholding* food and fluid unacceptable. Rebecca Dresser wrote, “It is possible that legal authorities would see the failure to help a debilitated patient eat and drink as more similar to [legally] prohibited active euthanasia than to permissible withholding and withdrawing of life-sustaining interventions" [61]. Clinically, if a physician’s order or a loved one’s behavior withholds food and fluid from patients, there will always be doubt regarding the ultimate cause of death since there are two possibilities: (A) withholding the vital substances of food and fluid, which could be characterized as euthanasia by omission, or (B) dementia destroyed the patient’s cognitive ability so severely that she could not recognize the items placed in front of her were food and fluid and/or could not coordinate moving her hands to put the offered food and fluid into her mouth. Either cause can lead to dying from her underlying disease.

A more acceptable request is to only *withdraw* assistance with assisted feeding, while *always offering* food and fluid by placing them within the patient’s reach. One could argue that the goal of this intervention is to determine if the loss of functioning is truly irreversible, and if not, the source of suffering. If so, then according to Brassington [62], “withdrawing life-sustaining treatment when death is not the intended outcome—and it may not be—is not euthanasia at all, passive or otherwise.”

16. Limited Ability to Combine Conditions Causing Only Moderate Suffering = LACOMS (0)

Opponents may dismiss directives as unacceptable if they list a condition that, *by itself*, causes only moderate suffering. Yet many planning principals still abhor such conditions—especially if they are simultaneously forced to endure a combination of such conditions.

Of those directives reviewed here, only the C&C Tool [30] allows planning principals to combine two or more of its listed conditions to fulfill the criteria to cease assisted feeding.

Some sources of suffering affect the patient’s loved ones. They may suffer from the empathy they feel for the suffering patient, as well as from the loss of no longer being able to interact with and enjoy the patient. For some planning principals, the combination of personal and loved one’s suffering—even if each is moderate—may combine to an intensity of suffering severe enough for them to want to die of their underlying disease. Yet none of the directives considered here include consideration of loved ones’ suffering.

17. Who is the Authority to Determine If It Is Time = WADIT (0)

Traditional directives have three options that can be selected “To *not* prolong life.” Since dementia patients are usually neither terminally ill nor in a state of severely impaired consciousness, they must select this secular version of the Catholic Principle of Proportionality [52, 53]: “If the likely risks and burdens of treatment would outweigh the expected benefits,” which the Uniform Healthcare Decisions Act [63] and many states have adopted [64].

While the Principle of Proportionality sets a laudably high goal in making end-of-life decisions, it does *not* designate *who* is the person authorized to weigh the benefits versus the burdens and harm. This is a common example: some family members want assisted feeding to continue because they hope the patient would briefly regain enough lucidity before she dies to say, “I love you. Goodbye.” Other family members argue this wish is selfish because it would prolong the patient’s suffering for a low probability event that would not likely provide the patient much, if any benefit. The Principle of Proportionality provides no guidance on *who* should make this decision. A prolonged conflict would not only render the directive ineffective and prolong dying; it could also threaten the family’s interpersonal relationships.

18. How Authority Determines If It Is Time = HADIT (0)

The Principle of Proportionality also provides no guidance on *how* to weigh one type of benefit versus a different type of harm or burden*.* Example: some family members argue the patient’s life is still worth living because she smiles about twice a week. Others argue that the longer she exists in advanced dementia, the more likely her survivors will remember her as a sick and dependent patient since the patient repeatedly and vehemently indicated she would loathe leaving tarnished memories. If the conflict were prolonged, the directive would be ineffective by prolonging dying.

19. Format Incompatible with Physicians Orders = FIPO (7)

Confucius taught, “He who chases two ducks catches neither.” All but one of the directives reviewed here (discussed below) uses *one form* for both planning principals and treating physicians. In striving to accomplish two functions, these directives may serve neither well. One function is to *educate*, *elicit*, and *memorialize* planning principals’ advance treatment decisions, where the form is a *patient decision aid* designed for use by planning principals. The other function is to *present* the resulting set of requests to future treating physicians, so they can write medical orders that accurately reflect their patient’s specific end-of-life requests. This second form can use medical jargon and include as much complexity as needed.

Ronald Dworkin warned, “The greatest insult to the sanctity of life is indifference or laziness in the face of its complexity” [65]. Yet Gaster and his primary care colleagues preferred one simple form for both planning principals and treating physicians [44]. They were frustrated by patients who failed to fulfill their promise to complete directives.^21^ They assumed their patients would be more likely to complete a simple “dementia-directive,” so they drafted a form that requires planning principals to check only one box. So, their form may be ineffective and lead to prolonged dying.

The C&C﻿ Tool [30] asks planning principals to first complete an online questionnaire; then, it uses these responses to generate a printout for physicians. But the C&C Tool is *not* directly compatible with the most widely used form to implement patients’ end-of-life requests: the POLST [66].^22^ This set of immediately actionable orders is designed to apply across all care settings, and health care providers are generally obligated to carry out these orders [67]. All POLSTs have three treatment options: (A) Full treatment; (B) Selective (or Limited) treatment designed to restore function but avoid burdensome treatments such as invasive ICU type of treatments; and (C) Comfort-focused treatment, to “allow natural death.” In contrast, the C&C Tool printout has four treatment options, which may make it difficult for physicians to transpose to three POLST orders. Worse, three of the four options refuse life-sustaining treatments, which allows opponents to allege the form is biased toward allowing a sooner death and thereby makes the directive unacceptable.^23^

Ferdinando Mirarchi led a series of research articles that showed the need for improving the accuracy with which emergency medical personnel and physicians respond to POLSTs and advance directives. His studies showed increased accuracy if planning principals recorded a short video that stated what interventions they did or did not want [68, 69]. For planning principals who anticipate advanced dementia, their video could explain why they want assisted feeding to cease, and then warn emergency medical personnel and physicians *not* to start an IV (which is usually automatic) since it would prolong their dying, which they do not want.

Omitting clarifying videos may lead to either premature or prolonged dying. While recording videos is not (yet) the standard of care, omitting such videos fulfills the definition of a flaw.

## Type IV flaws *omit* strategies designed to compel treating physicians to write orders that honor directive’s requests and to prevent third parties from sabotaging these orders

20. Strategies to Compel Orders by Treating Physicians = SCOTP (0)

Most state laws let physicians decline to comply with a directive’s request if the physician deems it “requires medically ineffective health care or health care contrary to generally accepted health care standards” [70] or “for reasons of conscience” [71]. These options are reasonable. The first strives to protect patients; the second respects health care providers.

But some physicians presume they know patients’ “best interest” *better* than the planning principals who diligently deliberated to make advance treatment decisions based on their personal values. Currently, some physicians have conflated the authority to *write* orders (that their state medical board granted) with the authority to *decide* what orders to write (that no entity has the power to grant). It is contrary to law and ethics for physicians to make decisions for their patients. Proxies/agents can quote probate code such as: “A health care provider…providing care to a patient shall…comply with an individual health care instruction of the patient…to the same extent as if the decision had been made by the patient while having capacity” [72]. Proxies/agents can engage consultants to warn treating health care providers that if they write orders that do *not* reflect patients’ *known* wishes *in good faith*, they can lose immunity and be sued criminally, civilly, and their licenses may be sanctioned administratively. If it is easy for physicians to *not* write orders that conform to planning principal's conditional request to cease assisted feeding, then patients may be forced to endure a prolonged dying.

21. Physicians Require Additional Clinical Criteria = PRACC (2)

If strategies are not in place to prevent health care providers from requiring additional clinical criteria before honoring a directive, the new directive that providers create by such additions may *not* reflect planning principal’s end-of-life goals. Adding criteria may reflect the hesitancy of health care providers to carry out the irreversible act of allowing a patient to die. Authorities^24^ who require patients to fulfill additional clinical criteria may be well-meaning and want to prevent premature dying; yet to our knowledge, no authority has ever provided clear and convincing evidence to prove they know *better* than planning principals, the best interest of a now-incapacitated dementia patient.

Below are four examples:

(A) Menzel [46] asked this rhetorical, morally provocative question: “If someone has a *clear* directive that food and water by mouth be withheld when she reaches a *certain stage*,^25^ but when she reaches this stage *still appreciates life and wants to eat and drink,* are we actually going to withhold food and water from her?” {Original emphasis.} The obvious answer, “No,” provided Menzel a segue to introduce his new philosophical concept: being able to appreciate living. In Menzel’s words: “Withholding food and fluid by mouth [depends]…on discerning *two* key points: *when* the dementia patient meets the triggering conditions in the person’s advance directive, *and when* the person’s continued stake in survival is sufficiently low*.*” {Emphasis added.}
(B) AMDA’s Policy A19 [56, 57] “recommend[ed] against implementing SED [stopping eating and drinking] by AD [advance directives] in those patients who still accept food and fluids, implementing instead, a policy of comfort feeding for those with advanced dementia…as long as the resident is not showing signs of distress^26^…or refusal^27^” [56, 57]. AMDA’s justification was (in part): “We not only show our patients that their current existence is less meaningful, we shorten their lives…[if] we implement SED by AD.”
(C) Ladislav Volicer et al. [73] (henceforth, *Volicer*) added this required criterion: physicians should wait to honor patients’ directives until patients *no longer request assisted feeding*. Volicer strived to respond to AMDA’s policy, as he assumed patients would stop requesting to be fed (or refuse feeding) well before they manifested distress from the act of feeding.
(D) Walsh wrote, “Having dementia is a cognitive transformative experience and…preference changes which result from this are legitimate and ought to be given moral weight in medical decision-making. This argument ought to encourage us to reduce our confidence in the moral weight of advance directives for dementia patients” [74]. Walsh’s philosophical concept can be used as a general justification by those who oppose honoring directives that request assisted feeding to cease. (It is beyond the scope of this article to discuss whether Walsh’s concept expands the perspective introduced by Dresser [75], whose work Walsh did not cite.)

Omitting strategies to let “Physicians Require Additional Clinical Criteria” may cause harm:

The three semi-fictional cases below include **Case I** and **Case II** that contrast a capacitated patient’s claim right to contemporaneous Voluntarily Stopping Eating and Drinking *versus* a physician’s unilateral refusal to honor a similar request that an incapacitated patient made in advance via his directive. **Case III** illustrates how knowing that such refusals are likely can lead to devastating harm.

*Case I* A 96-year-old woman is medically well, but lives alone, is lonely and bored with life. She outlived her husband and all close friends. Loss of hearing and sight has vastly diminished her ability to enjoy life. She decided to express her love for her offspring by the most tax-efficient way to transfer her assets: to die. Family members and professionals tried but failed to dissuade her from her plan. She has the legal right to die by Voluntarily Stopping Eating and Drinking (VSED). Her physicians are not legally permitted to impose their values; they cannot override her decision by force feeding, either by tube or mouth. This has been the law in California since *Bouvia* (1987) [76], and in the US since *Cruzan*﻿﻿ [6, 11] (1990). While she may be required to respond in a clinical interview to demonstrate she has capacity, she is otherwise *not* obligated to justify her reasons. Note that since she has capacity, her decision is *not* dependent on an advance request in a directive.

*Case II* A planning principal diligently and deliberatively created a directive that prioritized his lifelong critical interests over his future experiential interests, so that his life narrative could continue as best as possible after he lost capacity. His directive stated: “I want my estate to fund my grandchildren’s university education, to help them start their businesses, and especially to avoid ‘medical bankruptcy’ due the expense of my care [77]. I therefore request stopping all life-sustaining treatments that includes ceasing assisted feeding, when I no longer can enjoy life due to advanced dementia or another terminal illness.”

The treating physician adamantly refused to honor his directive. She explained: “Financial considerations should never determine the time of dying.” She insisted his request was contrary to generally accepted health care standards. Since she expressed her belief as if it were the professional norm instead of her personal conflict based on conscience, she was not obligated to make a reasonable attempt to transfer the patient to another, willing provider.

While well-meaning, the physician’s refusal dismissed the patient’s known lifelong values and imposed instead her own version of the patient’s “best interest.” This act of paternalism caused harm to the patient and his family members. His grandchildren were deprived of attending their top choice of universities; no funds were available to help start their new businesses; and, in less than three years, the family had to file for bankruptcy.

The patient suffered in two ways. He was prevented from sparing his family financial harms, and the ordeal left tarnished memories. His disease would forever be seen as the cause of his family’s great financial hardship. (While the patient could not be contemporaneously aware of this tragedy due to incapacity, society does not insist on awareness for the dead [78] as a prerequisite to avoid harm. Hence, society would similarly *not* insist on capacity for those who are either living or have died from advanced dementia.)

*Case III* After receiving the diagnosis of early dementia, a patient diligently researched the internet, made some phone calls, and reached this sad conclusion: he did *not* trust his future physicians would honor the requests in his directive and allow him to have a timely dying. This “Dementia Fear” caused him daily worry. Worse, he realized that if he waited too long to hasten his dying, he could become “stuck” for years in advanced dementia. While his decline in cognitive functioning was currently slow, he also feared an acute event such as a serious infection, head injury, or fall could lead to a sudden but devastating decline in physical or mental incapacity. If such an event led to his being admitted to a hospital or institution, its goldfish bowl environment would cause him to forever lose the opportunity to hasten his dying.

While he still enjoyed Dixieland jazz, international cuisine, and spending time with his children and grandchildren, he took definitive action to hasten his dying. He bought OTC items to decrease his thirst, asked his primary care physicians to prescribe a month’s supply of anti-anxiety medication, and then Voluntarily Stopped Eating and Drinking (VSED). His son remained by his side as he died peacefully.

Norman Cantor wisely commented, “Undertaking self-deliverance at an early stage of dementia entails the hazard of cutting short an existence that is still enjoyable (and might continue to be so for some unknown period)” [79]. Thus, omitting strategies designed to prevent Physicians [from] Requiring Additional Clinical Criteria (PRACC), can lead to the tragic harm of unnecessarily sacrificing years of reasonably good living. In other words, it can be patient-determined premature dying in response to intense worrying about prolonged dying.

The flaw, “Physicians Require Additional Clinical Criteria” (PRACC) may violate the four principles of bioethics [80, 81]:

Unilaterally changing the triggering criteria of advance directives for patients who cannot give their informed consent due to incapacity, but who previously made advance treatment decisions when they had capacity, does *not* respect patients’ right to self-determination. It thus violates the principle of *autonomy*.

The relentless downward trajectory of advanced dementia eventually renders patients unable to enjoy life. Prolonging the process of dying without improving patients’ lives violates the principle of *beneficence*.

Prolonging dying may increase and definitely prolongs various types of suffering—especially if not recognized or treated. This violates the principle of *non-maleficence*.

The cost of providing care for advanced dementia patients notoriously increases as patients’ ability to benefit decreases. Scarce medical resources could instead provide more benefit with less harm to other patients. Prolonging dying thus violates the principle of *social justice*.

22. Undermining Planning Principals’ Authority = UPPA (6)

Some who oppose orders to cease assisted feeding use a common conflict as a “conceptual wedge” to assert their values. Bioethicists have been debating how to resolve this conflict for three decades [82], so this article refers to it as the “classic conflict.”^28^ After the physician writes and implements an order to cease assisted feeding (which reflects the past planning principal’s preference), the currently incapacitated dementia patient nonverbally points to the food and fluid placed in front of her, then points to her mouth and grunts. Some patients can say, “Gimme,” but even nonverbal gestures may be clear enough for all to correctly interpret her desire for assisted feeding to resume. This is how the “classic conflict” emerges. It leads to debate over whether to honor the past or the current version of the patient’s wishes.

Opponents of the order to cease assisted feeding can use the “classic conflict” as a “conceptual wedge” to increase their power. For example, AMDA’s ethicists argued, “*We* either violate the entire concept of advance directive and practice an *injustice* against the person as they once were; or *we* refuse to feed our patient and practice an *injustice* against who they are now” [56, 57]. {Emphasis added.} While it is true that there is no *widely accepted* way to resolve the “classic conflict,” this does not give AMDA’s ethicists the right to recommend that AMDA providers should be appointed as the responsible parties to resolve the “classic conflict.” So, using the word “we” twice is  presumptuous and paternalistic.

A logical flaw was overlooked as AMDA’s ethicists argued for the adoption of Policy A19: the fallacy of bifurcation. The ethicists falsely presented the ethical dilemma as if only two options existed; that is, as if no other options were possible. Below, this article discusses other workable options. In addition, the ethicists committed the flaw of extrapolation, which makes their practice of paternalism more egregious. They categorically insisted that “The Society affirms the right of *all*…residents to receive comfort feeding until their behavior indicates refusal or distress.” {Emphasis added.}

### Four ways to overcome such formidable challenges: first a list, then more details

(A) Argue that “experts” are proposing a new clinical practice guideline while eschewing a rigorous developmental process;
(B) Explain how their guideline is flawed;
(C) Explain how their guideline violates prevailing law; and,
(D) Offer a strategy that can be implemented during advance care planning that is designed to prevent the “classic conflict” from emerging—to render these arguments moot.

(A) Eschewing a rigorous development process for a new clinical practice guideline

We are *not* aware of any currently available “dementia” directive that has undertaken the rigorous process of developing a new “clinical practice guideline” that “translates best evidence into best practice by emphasizing a logical sequence of key action statements supported by amplifying text, evidence profiles, and recommendation grades that link action to evidence” [83, 84]. This process can take a year or more. It involves collecting data from health care professionals and patients via focus groups and surveys, and running pilot trials that are analyzed to make improvements by an iterative process.

Empirical research in the field of advance care planning for late-stage dementia is rudimentary. Example: Santulli et al*.* held six workshops with 170 subjects; 40 completed their questionnaire; 27 answered “Yes” to, “Will you complete a directive for dementia for yourself?” Evidently, no follow-up was reported regarding what these few subjects actually did [85].

(B) The proposed recommendations are flawed

Menzel’s additions to planning principals' directives [46] have four﻿ flaws: (A) it provides *no* guidance on *how* clinicians can determine with sufficient medical certainty whether a patient’s stake in survival is, or is not, “sufficiently low” to be allowed to die; (B) the directive does not refer to a validated scale and suggest a cutoff score; and (C) Menzel does *not* specify *who* qualifies as a judge. (The issue of existence is broader than medicine, so non-physicians could qualify.)

Menzel’s fourth flaw is most worrisome: (D) He explicitly authorizes an unspecified third party to observe and interpret the behavior of nonverbal patients regarding whether patients do, or do not, value their own lives. Consider his example, “Sheri.” She “can hardly be said to value her survival or have a stake in her continued existence, for her cognitive deficits prevent her from even anticipating it. Survival—future existence—does not much matter to her now.”

Many moral leaders consider it wrong for one human being to judge whether the life of another human being is worth living. Implementing this practice could start a “slippery slope” that could (again) end in a humanitarian disaster [86].^29^

Regarding Volicer’s proposal [73], the question is, where shall the bar be placed for patient’s decisional capacity to honor their requests for assistance with *eating and drinking* (Volicer’s words)?^30^ Jaworska opined that if people with dementia *can value* activities and experiences in their lives, as demonstrated by *explaining* their choices, then their current decisions ought to override the requests in their directives [87]. 

The﻿ key question is: *how low* can a behavioral bar be placed, to fulfill Jaworska’s *value* requirement (for assisted feeding)? Does merely opening their mouths and swallowing what others put in suffice? Does smiling when fed? Does uttering, “Mmmm”? Those concerned about the patient’s well-being might also consider the balance: has the patient reached a condition that she previously judged would cause severe suffering?

Walsh’s article [74] and its 17 open-peer commentaries did *not* fully consider the goal of avoiding pain and suffering as a durable personal value. Yet every competent adult American has the *claim right* [88] to avoid severe suffering,^31^ and this right transcends loss of capacity. Advanced dementia patients are likely to experience more suffering than most health care providers currently appreciate since patients cannot complain and providers’ view of suffering may be narrow. Similarly, about 40% of patients presumed to be in the persistent vegetative state are misdiagnosed [89], so they are likely capable of experiencing pain and suffering.^32^

(III) Protocols that Undermine Planning Principals’ Authority (UPPA) may violate the spirit and letter of laws that discourage physicians from making treatment decisions on behalf of their patients. Consider four points:
  1. In many jurisdictions, treating physicians cannot legally serve as their patients’ proxies/agents. The intent of the law is to avoid a potential conflict of interest by reducing the power of treating physicians.
  2. If a conflict emerges between the health care instructions in a patient’s directive and the contemporaneous instructions of the currently acting proxy/agent, the instructional directive has legal priority (not the physicians’ judgment of “best interest”).
    Example: Cynthia Cardoza [90] sued her mother’s physicians for causing the patient to suffer; for denying the patient’s right to die with dignity; and for forcing Cynthia to experience severe emotional distress. The physicians/defendants claimed immunity by citing California Probate Code §4740 and stated they had complied “with the health care decision made by a *person whom they believed* was authorized to make this decision” (plaintiff’s brother). {Emphasis added.} But a California appeals court ruled that defendant physicians did *not act in good faith* because when these physicians ordered life-sustaining treatment and performed surgery, they had known that the patient’s *directive* had refused such curative treatment in advance.
  3. A semantic argument: since instructional directives have higher priority than proxies/agents who are legally designated by *durable* powers of attorney for healthcare decisions, instructional directives must also be *durable*.
  4. The Federal Patient Self-Determination Act of 1990 [91] states that providers may “*not condition* the provision of care or *otherwise discriminate* against an individual based on whether or not the individual has executed an advance directive.” Some interpret the PSDA as forbidding providers from discriminating by refusing to treat patients based on (otherwise) disagreeing with patients’ treatment decisions.^33^
(IV) A proposed strategy that is designed to prevent the classic conflict from emerging

To overcome this flaw, Undermining Planning Principals’ Authority (UPPA), strategies can be added that are designed to increase the power of proxies/agents. This topic is discussed next.

23. Undermining Proxies/Agents’ Power = UPAP (5)

AMDA’s Policy A19 [56, 57], does *not* advise physicians to meet their ethical and legal obligation to consider the substituted judgment of proxies/agents. Instead, A19 advises, “Although [our policy to refuse] may be an issue where common ground cannot be found with the health care proxy, the provider must *engage* with them and fully *explain* the rationale behind *the choice* to refuse to implement SED [stopping eating and drinking] by AD [advance directive].” {Emphasis added since using “engage” and “explain” is consistent with their position being intractable.} (This advice is repeated to indicate the challenge is formidable.)

To change physicians’ practice, a precedent-setting lawsuit or passing new laws may be required. Both can take much time and effort. Neither can guarantee success.

Professor Thaddeus Pope generally suggested using an *irrevocable Ulysses contract* to overcome the kind of challenges that Margaret Bentley experienced [92], which might overcome this flaw. This article recommends specific strategic details below.

Directives can present planning principals four or more choices of authorities to determine who should determine whether to honor the patient’s past or present expression of wishes:

(A) Their future treating physician. While this choice may at first seem to embrace AMDA’s Policy A19, the ethical difference is significant. This proposal asks patients if *they* want to make this choice voluntarily, which may depend on the patient trusting her physician’s promise to fulfill her directive’s requests. In contrast, A19 imposes this choice on incapacitated patients without their knowledge or consent. In the case of AMDA’s A19, the breach of trust is worse since the ethicists anticipated providers would have by then known that the requests in planning principals’ directives were the exact opposite.
(B) The planning principal’s “future demented selves,” as Volicer recommended. This choice is consistent with laws in many states that explicitly waive the capacity requirement for patients to receive life-sustaining treatment upon request [93].^34^ As reviewed above, many European countries allow patients to revoke their directives, regardless of capacity [37]. This option is a weak strategy for honoring controversial requests in patients’ directives since the “future demented self’s” behavior is unpredictable.
(C) Allow the proxy/agent 100% leeway to make this decision based on their substituted judgment that strives to consider patients’ values and what would be in patients’ best interest. Success is required at two levels. First, proxies/agents’ instructions must accurately reflect the patient’s wishes, which is problematic given the concordance problem. Second, proxies/agents must persuade the future treating health care provider to follow their controversial instructions. Opponents can challenge the proxy/agent’s motivation, or refuse to honor their instructions unless they provide clear and convincing evidence regarding what the patient would have wanted in the current condition. In addition, when the time comes to act, the first choice for the trusted proxy/agent may not be willing, available, or capable, and the alternate may not be as trusted, effective, or knowledgeable. This option may therefore promptly fail or lead to a prolonged conflict.
(D) The strongest strategy to make the memorialized requests in directives irrevocable is to empower proxies/agents to advocate the requests embodied in the directive. This requires two legal steps, First, planning principals must waive their future right to *apparently*^35^ object to their proxies/agents’ instructions. Second, the planning principal must sign a separate bilateral contract with each proxy/agent and alternate, whereby each proxy/agent promises to serve as a steadfast advocate to honor requests in the patient’s directive.

Note: planning principals can select other categories of individuals to consummate this bilateral “Ulysses contract.” They include community leaders, secular or religious counselors, or a specific individual such as a relative who, for example, is an attorney or physician.

While it takes time and effort to obtain additional signatures, this protocol may prevent a prolonged classic conflict from emerging. If so, opponents cannot use this conflict as a conceptual wedge to justify imposing additional clinical criteria on their patients.

24. False Interpretation of Behavior Observed = FIBO (3)

This flaw is discussed last for three reasons: it refers to previously presented examples; the flaw needs to be appreciated as being broadly applicable; and this flaw might inspire the humility needed to overcome paternalism.

This flaw is omitting strategies that challenge physicians and others from assuming *their interpretation* of an incapacitated patient’s nonverbal, observed behavior accurately reflects what the patient would want.

Jongsma wisely wrote that “changed behavior should *not* be taken at face value to indicate the development of a new value. Not being able to confirm earlier made decisions, or opposing what was important to this person, is part of the tragic decline and deprivation caused by dementia and should *not be understood* as an indication that these measures are now acceptable” [94].^36^ {Emphasis added.} A relevant example is a patient who passively opens her mouth to accept what others put in (sometimes, after much coaxing).

Misinterpreting patients’ behavior can lead to false negatives or false positives, the magnitudes of which are not known. As illustrated by the four examples below, misinterpretation can lead to either premature or prolonged dying. Definitions: a “false negative” means patients *only seem* to reject food and fluid but they really want to live; a “false positive” means patients *only seem* to want assisted feeding to continue but they really want to be allowed to die of their underlying disease.

*Case A’s nonverbal behavior* The patient stops requesting assistance with eating and drinking (Volicer’s criterion):

*Specific observation* The patient is silent or indifferent when offered assisted feeding.

*Interpretation* The patient wants to die.

*Action* Physician writes the order to cease assisted feeding. The patient dies.

*Reality:* *False Negative*. Dementia caused severe brain damage that limits the patient’s ability to nonverbally express her wish to be fed and to live.^37^ (Masked facies is also possible.)

*Conclusion* Misinterpretation led to *premature dying*.

*Case B’s nonverbal behavior* The patient refuses food and fluid (C&C’s Tool, End of Life Washington’s Instructions, AMDA's A19):

*Specific observation* Patient turns her head, clenches her mouth closed, or spits out what others put in her mouth.

*Interpretation* The patient wants to die.

*Action* Physician writes the order to cease assisted feeding. The patient dies.

*Reality:* *False Negative*. Unrecognized, untreated pain in the patient’s mouth or GI tract could have been the factor that led the patient to refuse food and to not chew or swallow.

*Conclusion* Misinterpretation led to *premature dying*﻿.

*Case C’s nonverbal behavior* The patient cooperates with assisted feeding without *distress* (Principle of Proportionality and AMDA's A19):

*Specific observation* Patient cooperates: she opens her mouth and swallows what others put in.

*Interpretation* Patient wants to live.

*Action* Physician writes the order to continue assisted feeding instead of honoring the request expressed in patients’ directive (which lists other sources of suffering). The physician forces the patient to wait until the physician can interpret the patient’s nonverbal behavior during assisted feeding as distress.

*Reality:* *False Positive*. The patient wants to die because she has reached a condition(s) that she previously judged would cause severe suffering from other sources and/or her disease burdens her loved ones. The patient seems to cooperate only because caregivers tap her lips or chin and thereby evoke her primitive reflex to open her mouth, and she then automatically swallows whatever they put in. She is merely continuing her lifelong habit of eating (now, being fed) three or four times a day. Dementia caused her to lose capacity, so she forgot and is unable to appreciate the negative consequences of continued assisted feeding.

*Conclusion* Misinterpretation led to *prolonged dying* and suffering—the exact opposite of what the planning principal wanted, that motivated her to complete her directive.﻿

*Case D’s nonverbal behavior* The currently incapacitated patient actively requests assisted feeding to *resume*, but this request conflicts with her previously expressed wish to cease assisted feeding, as memorialized in her directive. (This is the classic conflict.)

*Specific observation* The patient reached a condition(s) that she previously judged would cause severe enough suffering to cease assisted feeding. Her physician honored her directive by writing an order to cease assisted feeding. But after a few days, the patient pointed to the food and fluid placed in front of her, and then pointed to her mouth and grunted.

*Interpretation* Patient has changed her mind: she wants to be fed and to live. Her nonverbal behavior revokes her directive. The law errs on the side of life by explicitly *not* requiring her to have capacity to receive requested life-sustaining treatments.

*Action* Physician writes an order to resume assisted feeding that sustains the patient’s life.

*Reality:* *False Positive*. The patient wanted to die to avoid personal suffering and to spare her family members the burdens of her disease. Her behavior can be explained in several ways. She sees food and fluid in front of her but cannot understand why her caregivers have ceased assisted feeding. She is thirsty due to a temporary lapse in palliative care. She is hungry since ketogenesis has not yet decreased this symptom. Lack of capacity has led her to forget and to be unable to appreciate the negative consequences of continued assisted feeding that include her personal suffering and burdening her loved ones.

*Conclusion* Misinterpretation led to *prolonged dying* and suffering—the opposite of what the planning principal wanted, which was precisely why she completed her directive.

Conclusions about the flaw, False Interpretation of Behavior Observed (FIBO):

Physicians are *not* on solid clinical ground if they rely on interpreting their patients’ contemporaneous observed behavior as the basis for deciding whether to continue or to cease assisted feeding. Misinterpretation may cause premature dying for patients who want to live, or force patients to endure prolonged dying when they want to be allowed to die based on their severe suffering from other sources.

## Two cases where advance directives succeeded in fulfilling patient’s goals

The authors of this article optimistically believe that patients can attain their end-of-life goals without passing new laws or initiating lawsuits. Consider two (real-life) successes:

**Case IV** exemplifies a directive that was specific enough to be transposed to another disease.

*Case IV* A 71-year-old Australian man completed a directive whose clear instructions were to *not* provide him assisted feeding—even if he *appeared* willing and cooperative—if he reached a set of specific behavioral and functional conditions that commonly occur in advanced dementia. Several years later, he suddenly suffered a stroke. He seemed to be cooperating with assisted feeding, but his wife insisted he would certainly want assisted feeding to cease because his current conditions fulfilled the clinical behavior that his “dementia directive” described, even though his diagnosis was different.

His physicians honored his directive by writing an order to cease assisted feeding—despite prevailing law in Victoria that categorizes requests to cease feeding assistance as “a *values* directive…[that] can guide, but *not* mandate decisions.” This law explicitly allows clinicians *to continue* assisted feeding despite directives that request such assistance *to cease*. Physicians honored the patient’s directive because its requests were based on behavioral and functional descriptions—not on a stage or a condition of one specific illness. Also, this treatment instruction by the surrogate, his wife, was consistent [95].

The treating physicians/authors further noted: “Had [this patient] been admitted to an alternative health care setting in a different state, with different ethical or religious values, support for his refusal of feeding may have been regarded as unacceptable …[which] might have led to…conflict…and considerable distress for all involved.” The laws in Victoria, the hospital’s policy, and the treating physicians’ ethical views were also important. If any had been different, the outcome might not have been different.

**Case V** shows how a directive’s strategies can successfully overcome initial physician resistance.

*Case V* In 2009, “Charles” memorialized his living will by two telephonic clinical interviews that were burned into an audio CD. His physician recorded the opinion that his patient had decision-making capacity, despite being in early-stage dementia. The patient twice responded “Yes” and gave a reasonable explanation to the question, “If the process of dying by medical dehydration were uncomfortable, would you still want to cease assisted feeding, to avoid prolonged dying in advanced dementia?”

Seven years later, he reached an advanced stage of dementia. His proxy/agent/wife helped obtain his admission to an inpatient hospice, where she showed his treating physician the directive and asked him to honor its requests. The physician explained his refusal: “Ceasing assisted feeding destroys internal organs, is painful, and dying is *not* in Charles’ best interest.”

The wife consulted with the original advance care planning physician and then followed his advice. She presented the treating physician with: (1) a copy of the printed directive that included the comprehensive list of conditions the patient previously judged would cause severe enough suffering to want to be allowed to die; (2) the written opinion of the physician who conducted the advance care planning interview that Charles did possess capacity for this task; (3) the audio CD of the telephone interviews that included the patient’s consent to cease assisted feeding—even if the process caused discomfort; and (4) a copy of Linda Ganzini’s article [25], which reported the results of nurses’ observations and rating scales that showed on average, medical dehydration allows a “good” and “peaceful” dying. Finally (5), she invited the treating physician to contact the patient’s advance care planning physician to discuss these issues.

Two days later, the treating physician agreed to honor Charles’ wish to die by medical dehydration. He stated he became convinced “this is what Charles really wanted.” Dying took nine days and was peaceful [96].

The editors of the book that reported Charles’ case [97] commented: (A) “While unusual for traditional advance directives, extraordinary documentation is prudent for advance directives that request stopping eating and drinking”; (B) “Virtually all the pieces of an ideal directive for SED were in place; (C) [The case] illustrates [how ceasing assisted feeding] can become a more accepted option”; and they rhetorically asked, (D) “When a directive is as specific and its qualifying conditions are as clearly met as they were in Charles’ case…should clinicians follow the directive as ‘standard of care’”?

## Discussion

A measured comment:

Complex ethical conflicts are rarely resolved by simple solutions. A patient’s directive can state that reaching *any one* condition that was previously judged to cause severe suffering is enough to want assisted feeding to cease. Yet suppose a patient manifested these two conditions: (1) he could no longer enjoy interacting with family members or friends; but (2) he could still enjoy simple pleasures of life. So, when his family visited, he just sat there; but if staff placed headphones over his ears and played Dizzy Gillespie, the patient straightened up in his chair, smiled, and hummed along as he pretended to play drums with his silverware.

These two criteria could lead to conflict. Condition (1) supports an order to cease assisted feeding, but condition (2) supports an order to continue assisted feeding.

How can this conflict be resolved? Should the treating physician unilaterally make the ultimate existential decision? Should the currently acting proxy/agent and all alternates form a committee to discuss the options, and see if they can agree on what the patient would most likely have wanted? Should they wait until another condition emerges that causes severe enough suffering? Should the proxy/agent request an ethics committee consultation?

At a higher level of inquiry, is this conflict another flaw? If so, can it be avoided? One possibility is for directives to ask planning principals for their input on how to resolve such conflicts, if they occur, by choosing one of these choices (or writing another choice in): (1) “Always err on the side of life”; (2) “Always err on the side of reducing my suffering”; (3) “Always follow my proxies/agents’ consensus of substituted judgment.” (4) “Ask for, and then follow the recommendations of an ethics committee consultation.” (5) Ask a specific individual such as “My Attorney, Bernie.”^38^

### Related studies, including one that is likely the first study that assessed families’ attitudes regarding peaceful dying; and another that surveyed responses to the classic conflict

A Dutch study [98] was inspired by data that revealed only about half of family members felt their relative with dementia experienced a peaceful dying. The most common complaint about their loved ones’ quality of care was neglect and disrespect, and this correlated negatively with peaceful dying (although these authors did *not* ask family members if they considered physicians’ refusal to honor the requests in patients’ directives to be a devastating manifestation of neglect and disrespect).

Schoene-Seifert et al*.* [99] asked their German subjects if they would “follow a patient’s advance directive” that stated, “If I lose my capability to reliably recognize my family…I do not wish to be treated by CPR, with ventilators, artificial feeding (IV or tube), or antibiotics in case of life-threatening infections (e.g., pneumonia).”

The study revealed that “25% [of subjects] were *unwilling* to follow the patient’s…directive refusing…[treatment for pneumonia] in late-stage dementia.” The addition of a “meta-directive” that requested forgoing antibiotics, even if the patient appeared content, yielded the result that 16.3% would still *not* honor the request. Thus, two-thirds would *not* change their minds.^39^

Schoene-Seifert’s survey could be extended by asking subjects if they would be willing to follow a directive that requested “cease assisted feeding” (which is more controversial than refusing antibiotics), and if the criterion were “severe enough suffering” instead of “unable to reliably recognize my family.” Clearly, more studies are needed.

Availability of an adequate directive is only part of the solution to advance care planning. Barriers exist that must be overcome. For example, newly diagnosed early-stage dementia patients in Singapore were much less likely to acknowledge barriers to advance care planning than caregivers (10.5% versus 58%) [100].

## Conclusion

This conclusion has three parts: the limitations of this review; viewing paternalism as providers’ attempts to benefit themselves; and the responsibility of society to protect vulnerable nonverbal, incapacitated patients, which aspiration is not limited to those living with advanced dementia.

### **Limitations of this review:**

This article began by stating its critique would rely on evaluating directives *on their face*, by authors’ clinical experiences, and by literature reports. Empirical studies are needed to prove this statement: If directives avoid flaws and implement additional strategies, then treating physicians will be more likely to honor planning principals’ end-of-life requests. Yet to our knowledge, *no* study has yet compared the relative success of two or more directives for fulfilling planning principals’ wishes for any end-stage condition.^40^

Absent research, philosophy may encourage planning principals to invest additional effort into advance care planning—to decide what treatment they do or do not want and to inform others—which, like Pascal’s famous wager,^41^ is an inherent good—whether or not it turns out to be successful. Completing the task maximizes the probability of a timely dying as it minimizes the risk of premature or prolonged dying, but there are no guarantees in living or dying. Nevertheless, planning principals can benefit in three ways: (A) they can feel satisfaction from knowing they did their best in completing advance care planning; (B) they may feel enough confidence in their plan so they are *not* plagued by the Dementia Fear and do *not* consider orchestrating their hastened dying; and (C) they can offer loved ones the gift of knowing their end-of-life wishes and a plan that will likely be effective. Diligent strategic advance care planning can thus lead to lives that both planning principals and their loved ones can live more peacefully.

### Not all paternalistic flaws are created equal

Paternalistic actions can be noble, self-serving, or insulting. To illustrate, consider again the flaw, “Physicians Require Additional Clinical Criteria” (PRACC).

(A) Volicer’s goal [73] is *noble*. He strives to benefit patients who seem to “want to live” and enjoy being fed, but his intent is not to force them to wait as long as AMDA’s criteria would likely require. Yet Volicer’s protocol may still offend those who champion autonomy. They may argue it is *not* possible for patients to benefit from *any* treatment that others impose on them that they did *not* themselves request. Moreover, Volicer’s protocol can still cause harm: it can prolong patients’ suffering if they have reached a condition causing severe enough suffering.^42^
(B) The physician who refused to honor a directive based on financial considerations (**Case II**, above) can be viewed as *self-serving* if she was motivated by her personal and professional values and used her authority to override her patient’s values so that she could view *her* actions as moral. Worse, her refusal to write the requested orders caused considerable harm to both her patient and his family.
(C) AMDA’s ethicists’ Policy A19 [56, 57] is not only self-serving; it is also *insulting*. The proposed justification presumes that directives are “influenced by prejudice that, strangely enough, may be exercised against ones’ future self,” so that AMDA’s providers can view themselves as protectors of cognitively impaired, dependent patients living with dementia, and as heroes who prevented their lives from being *wrongly* (in *their* opinion) shortened. AMDA’s ethicists were fully aware that their “refusal to implement SED [stopping eating and drinking] by AD [advance directive] will no doubt be met with shock and a sense of betrayal by the family, and appropriately so.” The discrepancy between their statement which is an enlightened clinical perspective and their intractable maleficent behavioral recommendation based on the unsubstantiated allegation of prejudice is consistent with their position being arrogant and intractable, which reveals how formidable their challenge is.

#### **The plight of incapacitated, nonverbal patients:**

Regarding the legality of directives for late-stage dementia patients, Thaddeus Pope noted, “technical legality diverges from practical enforceability,” and, “There is a material difference between having a constitutional right and being able to exercise that right” [101]. In the wake of Policy A19, Jiska Cohen-Mansfield wrote, “Allowing persons with dementia the right to die would require policy and practice changes such as…to stop eating” [102].

In the future, laws may change, and lawsuits may set precedents that motivate malpractice insurance companies to educate their policy-holding physicians to act in accord with AMA’s Code of Ethics Opinion 2.20, which is worth repeating: “The social commitment of the physician is to sustain life and to relieve suffering. Where the performance of one duty conflicts with the other, the preferences of the patient should prevail” [21]. In the meantime, individuals will need to put extra effort into advance care planning: first, by striving to make their requests clear and convincing; second, by omitting flaws; and third, by including strategies designed to overcome common challenges.

Beyond the plight of advanced dementia patients is a more general question: Will society grant people *freedom to express* their end-of-life wishes, but then fail to protect them from those in power who disregard their wishes by imposing *their own* values on them, after they have become vulnerable, nonverbal, and incapacitated? Were society to permit those in power to refuse to honor patients’ autonomous right to self-determination, especially if motivated by their claim right to prevent prolonged, severe suffering for their “future demented selves,” such passivity in dealing with oppression could begin a slippery slope that could continue to disenfranchise many people including those who are medically well, but vulnerable in other ways, from expressing their fundamental, personal values.

Bioethics is the field of intellectual activity that grapples with conflicts that surround the beginning and ending of life. On June 24, 2022, the U S Supreme Court handed down a ruling [103] that allowed each state to pass laws to make abortion illegal—without exceptions. Pro-choice activists and end-of-life surrogate decision-makers have one fundamental goal in common: both vehemently advocate for self-determination. Yet they have this significant difference: Activists (by definition) are united and highly vocal. Surrogates, in contrast, typically feel isolated and powerless. A forthcoming article has the goal of overcoming this challenge.

## Data Availability

The manuscript does not contain any data. “Not applicable.”

## Appendix: Is a legal fight warranted for patients who use a flawed directive?

In 2020, the sponsoring organization for SADD [31] offered free legal assistance to help proxies/agents succeed in attaining patients’ end-of-life goals. The organization likely wanted to test the legality of their directive by obtaining a court ruling [104]. Such cases might be provoked by physicians who were reluctant to honor directives that requested assisted feeding to cease. A court ruling might reassure physicians they were on solid legal ground. Yet a winning lawsuit would likely only make compliance *permissible*, *not required*. The reason is that judges cannot and will not force physicians to write orders with which they disagree. Physicians and institutions can always invoke a conscientious objection clause.

Furthermore, lawsuits may fail because the SADD is flawed. As previously noted, a single flaw can provide opponents adequate justification to refuse to honor a directive. The SADD has three serious flaws and nine other flaws that are listed below:

(A) *Doesn’t Insist on Enough Suffering (DISES)* many patients “do not recognize family members, loved ones, and friends,” but still want to continue to live so they can enjoy “these nice people.”
(B) *Intervention Not Acceptable To Authorities (INATA)* the directive “asks that the scent of food not be present in my room,” which requires *withholding* food and fluid. Opponents can argue that withholding is euthanasia by omission and thus illegal.
(C) *Doesn’t Offer Workable Irrevocability (DOWI)* the addendum requests that “nothing I do be deemed a revocation of this Advance Directive.” Authorities may refuse to honor this meta-request, which may be ineffective without a bilateral Ulysses Contract.

Other flaws include:

Descriptions of Interventions and Conditions Not Understandable (DICNU). Grade Level is 16.

Fails to Ask for Verbal Explanations (FAVE).

Condition Reached; Is Still Content (CRISC).

Omits Conditions Often Dreaded (OCOD). It has only one condition.

Omits strategies designed to overcome the following challenges: Compel Orders by Treating Physicians (SCOTP); Physicians Require Additional Clinical Criteria (PRACC); Undermining Planning Principals’ Authority (UPPA); and, Undermining Proxies/Agents’ Power (UPAP). Finally, the supplement has the flaw, False Interpretation of Behavior Observed (FIBO).

## Abbreviations

AMA: American Medical Association
VSED: Voluntarily stopping eating and drinking
AD: Advance directive and SED is Stopping Eating and Drinking, which this article uses only when quoting others who use these abbreviations (but still includes the full terms)
AMDA: The Society for Post-Acute and Long-Term Care Medicine
NY Directive: End of Life Choices New York’s “Advance Directive for Receiving Oral Foods and Fluids in the Event of Dementia”
C&C Tool: Compassion & Choices’ “Dementia values and priorities tool”
SADD: Supplemental advance directive for dementia care
POLST: Physician orders for life-sustaining treatment
FAST: Functional assessment staging tool
DDD: Dartmouth dementia directive

## Footnotes

1 The estimate that nearly 2 out of 12 seniors will have dementia, and nearly 1 will be severe, used these U.S. data: 83.7 million people will be over 65; 13.8 million will have dementia; over 40% of these will be severe; and 90% of dementias are in those over 65. Future prevalence of dementia may be greater due to obesity and head trauma, and may be lower prevalence due to disproportionate COVID-19 deaths [3].

2 Terman, SA 2022. Abandon the three-decade U.S. tradition of asking surrogates for their substituted judgment to honor the end-of-life wishes of incapacitated patients, including patients living with advanced dementia. Preprint available at: https://ssrn.com/abstract=4175655 or http://dx.doi.org/10.2139/ssrn.4175688

3 Khachaturian, former director of the Office of Alzheimer Research at NIH, summarized 40 years of drug research by saying: “The lack of success in the search for a drug to improve the devastating symptoms of these chronic brain disorders has been one of modern medicine’s greatest frustrations with a failure rate of nearly 99.6%” [4]. Many experts consider the FDA’s recent approval of aducanumab not just contentious, but disgraceful [5].

4 Goals of care for nursing home patients living with dementia as stated by family were only 49% concordant with the goals perceived by nursing home﻿ staff [13].(Same as flipping a coin.)

5 Some experts in this field argue that suffering is not required for patients to want to avoid prolonged dying in advanced dementia. A broadened concept of suffering may resolve this conflict but is beyond the scope of this article.

6 Examples: Is it ethically “right” for directives to request assisted feeding to cease? Is assisted feeding basic care or medical treatment—or does it *not* make a difference? Should patients’ critical interests or experiential interests be prioritized?

7 The Robert Powell Center for Medical Ethics has a “Will to Live” document that can be downloaded, but the website recommends more specificity in describing end-stage conditions [22].

8 In the US, health care providers include physicians, nurse practitioners, physician assistants, and other clinicians who respond to medical orders, including emergency medical personnel first responders.

9 Patients in a persistent vegetative state, the minimally conscious state, other neurodegenerative diseases, and some post-stroke patients face similar challenges.

10 This article assigns a short title and a pronounceable conforming acronym to each flaw, although full terms are always used. Some acronyms may be useful as mnemonics that make searching terms easier. The numbers of other meanings attributed to the acronym according to one source [32] are given in parentheses.

11 The phrase “by mouth” clearly means caregivers will put food and fluid into patients’ mouths.

12 Testing [35] showed the NY Directive’s Reading Level was “difficult to read”; it and required a Reader’s Age of “21–22 years (college level).” Mueller et al. found the average readability of U.S. states’ directives was grade 12, but noted the National Work Group on Literacy and Health’s recommended Grade 5 or lower [36].

13 These words follow the link < ? > symbol: “Help me understand these levels of care.” There is no hint that this link contains additional, life-determining criteria [29].

14 In a private email on 2020–08-22.

15 Another example is the SADD [30] that requests: “I insist that nothing I do be deemed a revocation of this Advance Directive unless I revoke it in writing at a time when I have the mental capacity to make and revoke an advance directive.”

16 In an in-person communication, Guy Micco, MD, former Director of U. C. Berkeley’s Aging Center, opined, “There is a wide range of feelings regarding incontinence, and the combination of diapers, trained staff, and air freshening sprays can effectively allow incontinent patients to have a good quality of life.”.

17 In contrast, protocols for Medical Aid in Dying never require patients to ingest a lethal dose of medication as soon as they reach a condition, a stage, manifest a behavior, or even when the pharmacy delivers the prescription.

18 Ethicist Anthony Appiah gave this advice to a woman who married a man now living in advanced dementia: “The painful truth is that [your] status is ethically equivalent to that of a widow.” The New York Times, The Ethicists. https://www.nytimes.com/2021/08/31/magazine/alzheimers-ethics.html Accessed 11 Sept. 2021.

19 Gaster and his collaborators decided *not* to offer the option to refuse assisted feeding in their directive because, “The topic of nutrition can elicit strong emotions,” and, “providing room on an advance directive for someone to state that they would want an intervention that does not provide benefit creates potential for *inappropriate* medical care and conflict between clinicians and families” [6]. We would argue that first, at some point in the trajectory of progressive advanced dementia, all life-sustaining interventions will become inappropriate; second, discussing advance requests to cease assisted feeding can give them an opportunity to learn, decide, and memorialize their advance treatment decisions based on their personal values; and finally, it is clinically better to discuss an intervention that may elicit strong emotions during calm, diligent advance care planning than during a crisis after the patient has lost capacity.

20 Similarly, Menzel wrote, “Directives to *withhold* food and water by mouth is a realistic and morally justified way for people to control living into extended years of dementia” [46].

21 In a private email on 2020–01-07.

22 Almost all US states have a POLST program in some phase of development. Some forms have slightly different names. Physicians may honor a patient’s National POLST [67] even if their state has not formally adopted the national version of the form based on the generally accepted (but not universal) practice of reciprocity, the similarity of the forms, and the threat of losing immunity if a judge rules their refusal was not in good faith.

23 The C&C Tool specifically omits the “Selective (or Limited) Treatment” POLST option, which orders non-invasive, non-burdensome, non-IUC types of interventions to restore function (and may sustain life). Instead, it adds the order, “Keep me comfortable while stopping all treatments and withholding food and water so that I can die peacefully.”

24 Authorities may be in such fields as medicine, bioethics, law, religion, and philosophy.

25 Using “reaching a stage” instead of using behavioral criterion such as “severe enough suffering” is likely what created this ethical conundrum.

26 A reminder: AMDA’s ethicists limited the definition of suffering as distress caused by the act of feeding itself.

27 See the last flaw, False Interpretation of Behavior Observed (FIBO), which challenges the ability of providers to correctly interpret the meaning of their patients’ behaviors, including refusal of feeding.

28 Also referred to as the “then-self” versus “now-self” conflict; and the “past-directive-vs-present-interest” conflict.

29 Sulmasy warned and explained how this practice could begin a dangerous slippery slope. The Nazi T4 experiment began as a benevolent attempt by physicians and attorneys to decide what is in the best interest of incapacitated patients, but ended in a nightmarish affront to humanity.

30 Volicer’s use of the term “to eat and drink” seems inappropriate since “feeders” (as they are sometimes referred to) are not enjoying a mutual social experience; instead, they are carrying out their job to fulfill physicians’ orders. The term, “To orally feed and hydrate,” seems more appropriate.

31 Claim rights, which may be positive or negative, allow holders to demand others act to fulfill their right [88].

32 A patient, who had been in an unresponsive vegetative state for 14 years, began to moan (in pain) and then gave birth. Only then did nursing home staff realize she was pregnant and must have been the victim of rape [32]. https://www.thesun.co.uk/news/8149636/woman-coma-vegetative-state-birth-rape-arizona/ Accessed 15 May 2022.

33 Providers who claim a conflict of conscience are obligated to make a reasonable attempt to refer their patients to another provider. (Reasonable is *not* defined.).

34 Pennsylvania’s statute is: “Regardless of the principal’s mental or physical capacity, a principal may countermand a health care decision made by the principal’s health care representative that would withhold or withdraw life-sustaining treatment at any time and in any manner by personally informing the attending physician.” [93]

35 “Apparently” means that the incapacitated patient behaviorally seems to object to the instructions of proxy/agent but her impairment in memory and judgment has led to her lacking knowledge and appreciation of the adverse consequences of continuing assisted feeding.

36 To our knowledge, Jongsma’s wise advice is one of few published that can be viewed as focusing on clinicians’ arrogance in believing that their interpretations of what they observe are (always) correct.

37 This is a specific example of Jongsma’s challenge [94].

38 This is the title of a humorous jazz tune composed by Dave Frishberg.

39 There was no significant difference among these four groups: physicians, nurses, next of kin who had experience with dementia, and dementia-inexperienced adults.

40 Pubmed and Google Scholar searches were conducted 30 Aug 2021, although admittedly this is a negative result.

41 Even if God and Heaven do *not* exist, a life lived morally is a good itself.

42 Note that Volicer [73] assumes but offers no evidence that the duration of time until death will be shorter.
