# Correction to: Flaws in advance directives that request withdrawing assisted feeding in late-stage dementia may cause premature or prolonged dying

**DOI:** 10.1186/s12910-022-00850-4

**Published:** 2022-11-16

**Authors:** Stanley A. Terman, Karl E. Steinberg, Nathaniel Hinerman

**Affiliations:** 1 Caring Advocates, Sausalito, CA USA; 2 California State University Shiley Haynes Institute for Palliative Care, San Marcos, CA USA; 3 Department of Theology and Religious Studies and School of Nursing and Health Professions, University of San Francisco, San Francisco, USA

## Correction to: BMC Medical Ethics (2022) 23:100 10.1186/s12910-022-00831-7

Following publication of the original article [1], the author would like to change the text from:

I want to be offered food and fluids by mouth. I want to be kept clean and warm.

to.

I want to be offered food and fluids by mouth if it is safe for me to eat and drink. I want to be kept clean and warm.

The original article has been corrected.
